# Impact of Intrinsic Capacity on 5-year Mortality of Older Patients With Cardiovascular Disease

**DOI:** 10.31083/RCM37477

**Published:** 2025-08-28

**Authors:** Yuhao Wan, Wenzheng Li, Junpeng Liu, Ke Chai, Hua Wang, Jiefu Yang

**Affiliations:** ^1^Department of Cardiology, Beijing Hospital, National Center of Gerontology, Institute of Geriatric Medicine, 100730 Beijing, China; ^2^Beijing Hospital, National Center of Gerontology, Institute of Geriatric Medicine, Chinese Academy of Medical Sciences & Peking Union Medical College, 100730 Beijing, China

**Keywords:** intrinsic capacity, elderly people, cardiovascular disease, prognosis

## Abstract

**Background:**

Intrinsic capacity (IC) is defined as the combination of all physical and mental (including psychosocial) capacities that an individual can rely on at any given time. Previous studies have shown that a decline in IC is linked to an increased mortality rate. Thus, this study aimed to evaluate the impact of IC on the 5-year mortality of older people with cardiovascular disease.

**Methods:**

This was a prospective cohort study conducted at a tertiary-level A hospital in China between September 2018 and April 2019, with a follow-up period of 5 years. We applied a proposed IC score to assess the baseline IC of each participant. The primary clinical outcome was 5-year all-cause mortality.

**Results:**

A total of 524 older patients (mean age, 75.2 ± 6.5 years; 51.7% men) were enrolled from the cardiology ward. A total of 86 patients (16.5%) experienced all-cause mortality over the 5-year follow-up period. Compared with the survival group, patients in the mortality group were older (81.1 ± 5.7 vs. 74.0 ± 6.0; *p* < 0.01), showed a higher male proportion (61.6% vs. 49.8%; *p* = 0.04), had a lower intrinsic score [7.0 (6.0, 8.0) vs. 8.0 (7.0, 9.0); *p* < 0.01], and a higher prevalence rates of atrial fibrillation or atrial flutter (34.9% vs. 20.1%; *p* < 0.01), heart failure (44.2% vs.11.2%; *p* < 0.01), diabetes (48.8% vs. 33.1%; *p* < 0.01), and chronic kidney disease (19.8% vs. 4.3%; *p* < 0.01). After adjusting for covariates, multivariate Cox regression showed that the IC score was associated with a lower hazard ratio of 5-year all-cause mortality (hazard ratio (HR) = 0.79, 95% confidence interval (CI): 0.69–0.92, *p* < 0.01).

**Conclusions:**

Among these older aged patients with cardiovascular disease, the IC score is independently associated with 5-year all-cause mortality, with a lower IC score indicating a poorer prognosis.

**Clinical Trial Registration:**

ChiCTR1800017204; date of registration: 07/18/2018. URL: https://www.chictr.org.cn/showproj.html?proj=28931.

## 1. Introduction

Global aging is accelerating, with an estimated 21% of the population projected to be over 65 by 2050 [1]. An increased longevity is not always accompanied by good health [2], and the aging of the world population will result in high medical costs and significant healthcare burdens [3]. In response to this challenge, The World Health Organization defined ‘intrinsic capacity’ (IC) as an indicator of healthy aging [4]. IC is defined as the combination of all physical and mental (including psychosocial) capacities that an individual can rely on at any given time [4]. Previous studies have shown that a decline in IC is linked to an increased mortality rate [5, 6, 7]. There is general agreement on the various dimensions of IC, which include locomotion, vitality, sensory, cognition, and psychological aspects [8]. Nevertheless, there is significant variation in the methods used to assess each of these five dimensions across different studies, and a unified approach for evaluating IC has yet to be agreed upon [9]. López-Ortiz S *et al*. [9] proposed a standardization for assessing each dimension of IC, employing a global scale ranging from 0 (worst) to 10 (best). Currently, IC has not been widely applied in elderly cardiovascular patients. This study applied this scoring system for constructing an IC score and explore the association between the IC score and 5-year all-cause mortality in elderly patients with cardiovascular.

## 2. Materials and Methods

### 2.1 Study Population

**Fig. 1.**
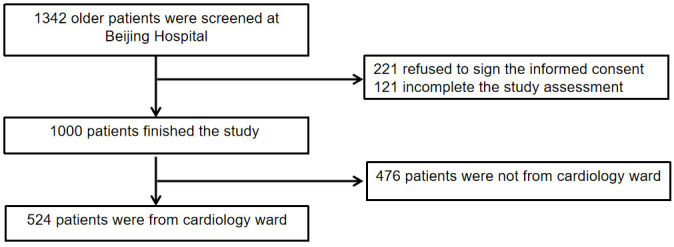
**Flow chart of patient selection**.

The data for this study were derived from a prospective observational cohort study conducted in China (Trial registration: ChiCTR1800017204). Elderly patients aged 65 years and older, admitted to Beijing Hospital between September 2018 and April 2019, were recruited. The study received approval from the Ethics Committee of Beijing Hospital (No. 2018BJYYEC-121-02). The criteria for inclusion were as follows: (1) Individuals aged 65 years or older, hospitalized due to cardiovascular illnesses; (2) Voluntary participation in this study with signed informed consent. The exclusion criteria included: (1) Patients unable of completing the thorough geriatric assessment due to significant cognitive impairment, hearing loss, or other problems; (2) Patients with acute exacerbation of chronic obstructive pulmonary disease (COPD); (3) Patients with end-stage renal disease undergoing dialysis; (4) Refusal to sign informed consent. A total of 524 elderly patients from the cardiology ward were included in the study. The participant inclusion process is illustrated in Fig. 1.

### 2.2 Information Collection

In this study, baseline data were gathered from the electronic health records of the patients. This data included information such as demographic information (e.g., age, gender), hospitalization details, medical conditions, physical examination results, laboratory test values, and other relevant health information.

### 2.3 Construction of IC Score

The IC score is a measure designed to assess healthy aging, with a range from 0 (representing the worst possible IC) to 10 (representing the highest possible IC). The score equally weighs five dimensions that are critical to an individual’s overall health and functional status. Five Dimensions were: cognition, vitality, sensory, psychological and locomotion. For each of these five dimensions, the IC score is stratified within a 0–2 range, based on the level of function impairment in each dimension: 0 = function significant loss, 1 = function decline, 2 = function stable. Followings are the detailed scoring criteria:

① Cognition (0–2 points): we applied mini-mental state examination (MMSE) to assess patients’ cognition dimension [10]. A MMSE score of 27–30, represents normal cognitive function, scoring 2 points in this dimension; a MMSE score of 10–26, represents mild to moderate cognitive impairment, scoring 1 point in this dimension; a MMSE score of 0–9, represents moderate to severe cognitive impairment, scoring 0 points in this dimension.

② Vitality (0–2 points): we applied the mini nutritional assessment - short form (MNA-SF) to assess patients’ vitality dimension [11]. A MNA-SF score of 12–14, represents normal nutritional status, scoring 2 points in this dimension; a MNA-SF score of 8–11, represents a risk of malnutrition, scoring 1 point in this dimension; a MNA-SF score of 0–7, represents malnutrition, scoring 0 points in this dimension.

③ Sensory (0–2 points): we applied two self-reported question to assess patients’ sensory dimension. “Do you have any difficulty with your hearing?”, response: yes = 1 point; no = 0 point. “Do you have any difficulty with your vision?”, response: yes = 1 point; no = 0 point. The combined scores from the two questions constitute the final score for this dimension.

④ Psychological (0–2 points): we concurrently applied the geriatric depression scale - 5 items (GDS-5) and the hospital anxiety and depression scale-anxiety subscale (HADS-A) to assess patients’ psychological dimension [12, 13]. A GDS-5 score of 0–1, represents no depression, scoring 1 point; a GDS-5 score of 2–5, represents a likelihood of depression, scoring 0 point. A HADS-A score of 0–7, represents no anxiety, scoring 1 point; a HADS-A score of 8–21, represents borderline or significant anxiety, scoring 0 point. The combined scores from the two scales constitute the final score for this dimension.

⑤ Locomotion (0–2 points): we applied the short physical performance battery (SPPB) to assess patients’ locomotion dimension [14]. A SPPB score of 10–12, represents robustness, scoring 2 points in this dimension; a SPPB score of 3–9, represents possible sarcopenia, scoring 1 point in this dimension; a SPPB score of 0–2, represents sarcopenia and cachexia, scoring 0 point in this dimension.

### 2.4 Variable Definition

Age, creatinine, and ejection fraction score (ACEF score) [15]: The ACEF score, which stands for age, creatinine, and ejection Fraction, is a simplified risk stratification tool originally developed to predict operative mortality in patients undergoing elective cardiac surgery. It is calculated using the following formula: Age (years)/Left ventricular ejection fraction (LVEF) (%) + 1 (if serum creatinine >2.0 mg/dL).

Atrial fibrillation (AF)/Atrial flutter (AFL): AF and AFL are common supraventricular arrhythmias characterized by disorganized or rapid atrial electrical activity, leading to irregular or rapid ventricular response. Both persistent and paroxysmal AF/AFL were included in this study.

Cancer history: This variable is extracted from the discharge diagnoses of the participants. Individuals with current malignant neoplasms were not included in this study.

Coronary artery disease (CAD): CAD is a condition characterized by atherosclerotic narrowing or blockage of the coronary arteries. This variable is extracted from the discharge diagnoses of the participants.

Chronic kidney disease (CKD): The estimated glomerular filtration rate (eGFR) <60 mL/min/1.73 m^2^ was defined as CKD, eGFR was calculated using the CKD Epidemiology Collaboration equation [16]. This study did not include patients with end-stage renal disease receiving hemodialysis treatment.

COPD: is a progressive respiratory disorder characterized by persistent airflow limitation that is not fully reversible. This variable is extracted from the discharge diagnoses of the participants. Participants with acute exacerbation of COPD were excluded from this study.

Diabetes: Diabetes is a chronic metabolic disorder characterized by hyperglycemia resulting from defects in insulin secretion, insulin action, or both. This variable is extracted from the discharge diagnoses of the participants. The specific types of diabetes were not differentiated in this study.

Heart failure (HF): HF is a clinical syndrome characterized by impaired cardiac function resulting in inadequate perfusion to meet the metabolic demands of the body. This variable is extracted from the discharge diagnoses of the participants.

Hypertension (HTN): HTN is a chronic medical condition characterized by persistently elevated arterial blood pressure. This variable is extracted from the discharge diagnoses of the participants.

Polypharmacy [17]: Polypharmacy was defined as the presence of ≥7 different drugs taken by a patient at discharge.

Smoking: Including participants who are current smokers or have a history of smoking.

### 2.5 Study End Point

The primary outcome of this study was the rate of all-cause mortality over a period of five years. Clinical follow-ups were routinely performed via phone annually. If unable to contact the patient or their family, the patient’s medical records were used to determine their survival status.

### 2.6 Statistical Analysis

Continuous baseline variables were summarized using either the mean with standard deviation (SD) or the median with interquartile range (IQR), based on the data distribution. Categorical variables were described as frequencies and percentages. Student’s *t*-test was used to compare normally distributed continuous variables between groups, while the Mann–Whitney U test was employed for continuous variables that were not normally distributed. For categorical variables, the Chi-square test was utilized to compare data between groups. We used receiver operating characteristics (ROC) curve analysis to obtain the optimal cut-off point of IC score to predict 5-year all-cause mortality according to maximal Youden index. In this study, univariable and multivariable Cox proportional hazards analyses were conducted to identify factors associated with all-cause mortality over the 5-year period. Kaplan-Meier analysis was used to estimate the cumulative incidence of the primary endpoint, and log-rank tests to analyze for significant differences. In this study, variance inflation factor (VIF) values were computed for all predictors. A stepwise elimination procedure was applied, removing the variable with the highest VIF in each iteration until all remaining variables had VIF values below 5. To identify independent predictors of survival, Cox proportional hazards regression analysis was performed. A stepwise variable selection procedure based on Akaike’s Information Criterion (AIC) was applied using backward elimination. Starting from the full model, variables were sequentially removed to minimize the AIC value, aiming to achieve a best model. The final multivariable Cox model included covariates retained after stepwise selection. We also employed Cox proportional hazards regression models to conduct subgroup analyses on the association between IC score and 5-year all-cause mortality. Subgroups were classified by age, sex and comorbidities. To evaluate the predictive value of the IC score, we compared it with the ACEF score by assessing improvements in discriminative ability using the delta C-index, Integrated Discrimination Improvement (IDI), and Net Reclassification Index (NRI). In this study, all statistical tests were two-tailed, and a *p* value of <0.05 was considered statistically significant. All analyses were conducted using R software (version 4.2.2; R Core Team, Vienna, Austria).

## 3. Results

### 3.1 Baseline Characteristics

**Fig. 2.**
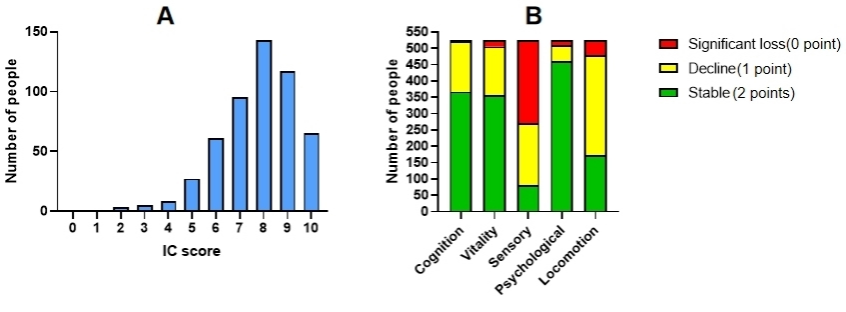
**The distribution of patients’ IC score and the score of the five IC dimensions**. (A) Overall IC score distribution, (B) Distribution of scores across the five dimensions. Abbreviation: IC, Intrinsic capacity.

Among 524 participants, the mean age was 75.2 ± 6.5 years old, and 51.7% were men. The median IC score was 8.0 (7.0, 9.0). The distribution of patients’ IC score and the score of the five IC dimensions are presented in Fig. 2, with the sensory and locomotion dimensions showing relatively high rates of function decline (1 point) or significant loss (0 point). The participants were divided into 2 groups (mortality group and survival group). Baseline characteristics comparison between two groups are shown in Table 1. Compared with the survival group at baseline, patients in the mortality group were older (81.1 ± 5.7 vs. 74.0 ± 6.0, *p* < 0.01), showed a higher male proportion (61.6% vs. 49.8%, *p* = 0.04), had a lower IC [7.0 (6.0, 8.0) vs. 8.0 (7.0, 9.0), *p* < 0.01], and a higher prevalence rates of AF or AFL (34.9% vs. 20.1, *p* < 0.01), HF (44.2% vs. 11.2%, *p* < 0.01), diabetes (48.8% vs. 33.1%, *p* < 0.01) and CKD (19.8% vs. 4.3%, *p* < 0.01). Moreover, patients in the mortality group had lower hemoglobin (Hb) value, lower ALB value, lower LVEF, higher uric acid value, higher ACEF score and higher n-terminal pro-B-type natriuretic peptide (NT-proBNP) value.

**Table 1.** **Baseline patients characteristics**.

	Overall	Survival group	Mortality group	*p* value
	n = 524	n = 438 (83.5%)	n = 86 (16.5%)	
Age, y	75.2 ± 6.5	74.0 ± 6.0	81.1 ± 5.7	<0.01
Male	271 (51.7)	218 (49.8)	53 (61.6)	0.04
Smoking	168 (32.1)	135 (30.8)	33 (38.4)	0.17
BMI, kg/m^2^	25.2 ± 3.4	25.4 ± 3.3	24.2 ± 3.7	<0.01
Heart rate, bpm	70.6 ± 13.5	70.1 ± 12.9	72.8 ± 16.1	0.10
SBP, mmHg	133.5 ± 17.0	133.7 ± 16.4	132.2 ± 19.8	0.43
DBP, mmHg	74.7 ± 9.8	74.9 ± 9.3	73.4 ± 11.8	0.19
AF/AFL	118 (22.5)	88 (20.1)	30 (34.9)	<0.01
CAD	359 (68.5)	301 (68.7)	58 (67.4)	0.82
HTN	383 (73.1)	313 (71.5)	70 (81.4)	0.06
CKD	36 (6.7)	19 (4.3)	17 (19.8)	<0.01
HF	87 (16.6)	49 (11.2)	38 (44.2)	<0.01
COPD	30 (5.7)	23 (5.3)	7 (8.1)	0.31
Stroke/TIA	90 (17.2)	71 (16.2)	19 (22.1)	0.19
Diabetes	187 (35.7)	145 (33.1)	42 (48.8)	<0.01
Cancer history	42 (8.0)	30 (6.8)	12 (14.0)	0.03
Polypharmacy	236 (45.0)	189 (43.2)	47 (54.7)	0.05
Hb, g/L	128.7 ± 15.7	130.1 ± 14.6	121.3 ± 19.0	<0.01
ALB, g/L	39.8 ± 3.0	40.1 ± 2.8	38.2 ± 3.5	<0.01
TB, µmol/L	10.7 (8.4, 14.1)	10.7 (8.4, 14.0)	11.1 (8.0, 15.5)	0.68
Uric acid, µmol/L	324.0 (267.0, 390.0)	319.0 (264.0, 378.3)	359.0 (300.5, 420.5)	<0.01
LDL-C, mmol/L	2.2 ± 0.7	2.2 ± 0.7	2.0 ± 0.7	0.06
Scr, µmol/L	70.0 (59.0, 86.0)	68.0 (59.0, 82.0)	86.0 (72.0, 115.0)	<0.01
LVEF, %	63.0 (60.0, 65.0)	65.0 (60.0, 65.0)	60.0 (55.0, 65.0)	<0.01
NT-proBNP, pg/mL	172.6 (78.5, 493.9)	150.9 (71.9, 350.6)	633.6 (216.6, 1537.0)	<0.01
ACEF score	1.21 (1.11, 1.34)	1.19 (1.10, 1.29)	1.45 (1.28, 1.72)	<0.01
IC score	8.0 (7.0, 9.0)	8.0 (7.0, 9.0)	7.0 (6.0, 8.0)	<0.01

Abbreviations: ACEF score, age, creatinine, and ejection fraction score; BMI, body mass index; SBP, systolic blood pressure; DBP, diastolic blood pressure; AF, atrial fibrillation; AFL, atrial flutter; CAD, coronary artery disease; HTN, hypertension; CKD, chronic kidney disease; HF, heart failure; COPD, chronic obstructive pulmonary disease; TIA, transient ischemic attack; Hb, hemoglobin; ALB, albumin; TB, total bilirubin; Scr, serum creatinine; LDL-C, low-density lipoprotein cholesterol; LVEF, left ventricular ejection fraction; NT-proBNP, n-terminal pro-B-type natriuretic peptide.

### 3.2 Clinical Outcomes

The 5-year survival status of all 524 participants was available, all-cause mortality occurred in 86 patients (16.5%). Univariable Cox regression analysis (Table 2) showed that IC score was a protect factor of all-cause mortality (HR: 0.62; 95% CI: 0.56–0.70, *p* < 0.01). Additionally, age, AF/AFL, HTN, CKD, HF, diabetes, cancer history, polypharmacy, lower BMI, lower Hb values, lower ALB values, lower LVEF values, higher uric acid values and higher LogNT-proBNP values were statistically significant risk factors.

**Table 2.** **Univariable cox regression analysis for 5-year all-cause mortality**.

Variables	Univariable analysis		
	HR	95% CI	*p* value
Age	1.17	1.13–1.21	<0.01
Male	1.53	0.99–2.37	0.05
IC score	0.62	0.56–0.70	<0.01
BMI	0.90	0.84–0.96	0.01
Heart rate	1.01	0.99–1.03	0.08
AF/AFL	1.92	1.23–2.99	0.04
CAD	0.95	0.60–1.49	0.82
HTN	1.74	1.01–2.99	0.04
CKD	4.55	2.67–7.73	<0.01
HF	4.90	3.20–7.51	<0.01
Diabetes	1.82	1.20–2.78	<0.01
Cancer history	2.09	1.14–3.85	0.02
Polypharmacy	1.53	1.00–2.34	0.05
Hb	0.97	0.95–0.98	<0.01
ALB	0.83	0.77–0.88	<0.01
LDL-C	0.74	0.54–1.01	0.06
Uric acid	1.01	1.00–1.01	<0.01
LVEF	0.95	0.93–0.97	<0.01
Log NT-proBNP	4.26	2.80–6.48	<0.01

Abbreviations: CI, confidence interval; HR, hazard ratio; Log NT-proBNP, logarithm n-terminal pro-B-type natriuretic peptide.

Variables were ultimately selected for inclusion in the multivariable Cox regression model based on initial criteria, which included variables with a *p* value < 0.10 in the univariate Cox regression analysis. Stepwise regression was then performed, and the AIC was calculated to select the optimal model. The initial variables included in the model were IC score, age, male, Heart rate, BMI, AF/AFL, HTN, diabetes, cancer history, CKD, HF, polypharmacy, Hb, uric acid, ALB, LDL-C, LVEF, and NT-proBNP. The initial AIC was 930.29. After stepwise regression, the final model included eleven variables: IC score, age, male, HTN, HF, diabetes, CKD, BMI, uric acid, Hb and LVEF, with an AIC of 915.60 and a C-index of 0.844. After adjusting for covariates, multivariate Cox regression showed that IC score was independently associated with a lower hazard ratio of 5-year all-cause mortality (HR = 0.79, 95% CI: 0.69–0.92, *p* < 0.01) (Table 3).

**Table 3.** **Multivariate cox regression analyses for 5-year all-cause mortality**.

Variables	Multivariable analysis		
	HR	95% CI	*p* value
IC score	0.79	0.69–0.92	<0.01
HF	1.72	0.99–2.99	0.05
Age	1.13	1.09–1.17	<0.01
Male	1.64	1.01–2.60	0.04
HTN	1.55	0.87–2.75	0.14
CKD	2.33	1.28–4.27	0.01
Diabetes	1.47	0.90–2.37	0.12
BMI	0.95	0.88–1.02	0.14
Uric acid	1.00	0.99–1.00	0.13
Hb	0.98	0.97–0.99	0.03
LVEF	0.98	0.95–0.99	0.05

**Fig. 3.**
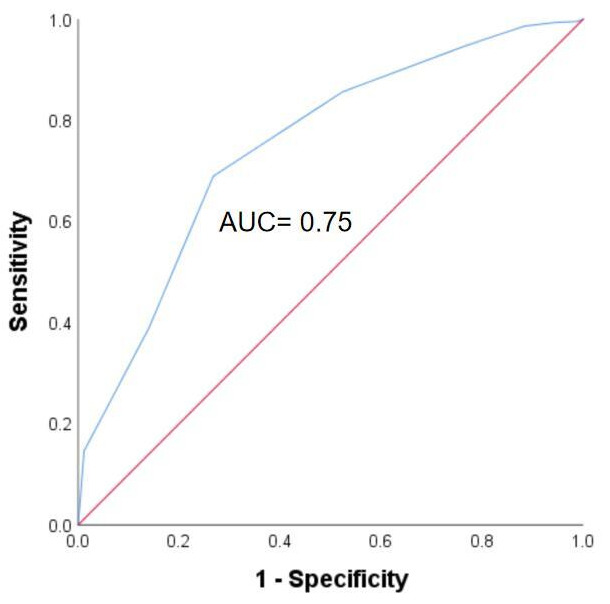
**Receiver operating characteristics curves for predicting 5-year mortality**. Abbreviation: AUC, Area under the curve.

**Fig. 4.**
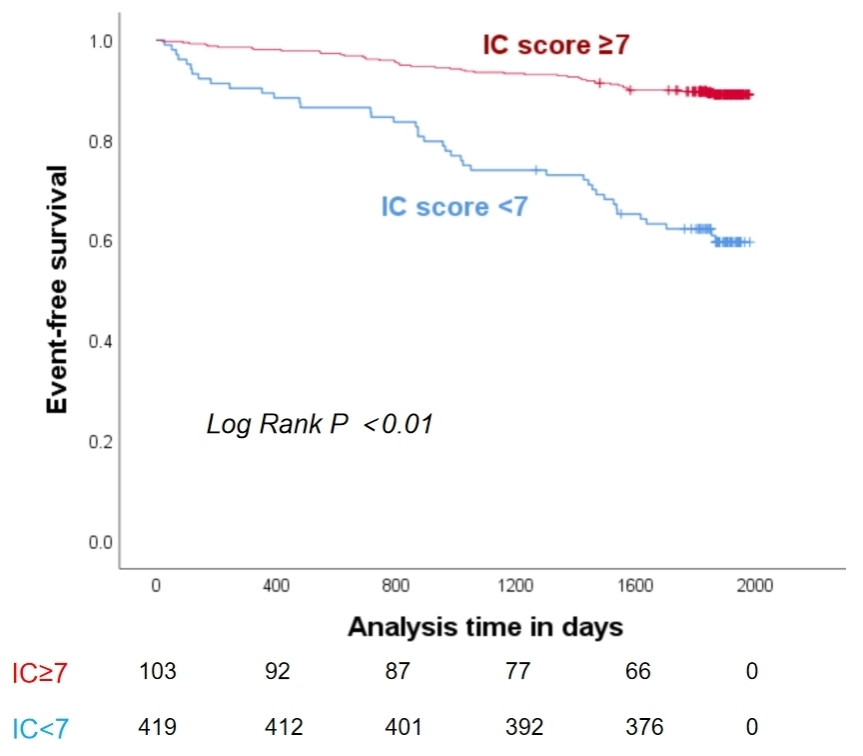
**Kaplan-Meier curve for the association between IC score and 5-year all-cause mortality**.

ROC analysis was used to evaluate to obtain the optimal cut-off point of IC score to predict 5-year all-cause mortality according to maximal Youden index (Fig. 3). IC score = 7 points (rounding 6.5) was the optimal cut-off point to predict 5-year all-cause mortality. Kaplan-Meier survival curve showed IC score <7 points has significantly higher all-cause mortality survival rate (log-rank *p* < 0.01) within 5 year (Fig. 4).

### 3.3 Subgroup Analysis

**Fig. 5.**
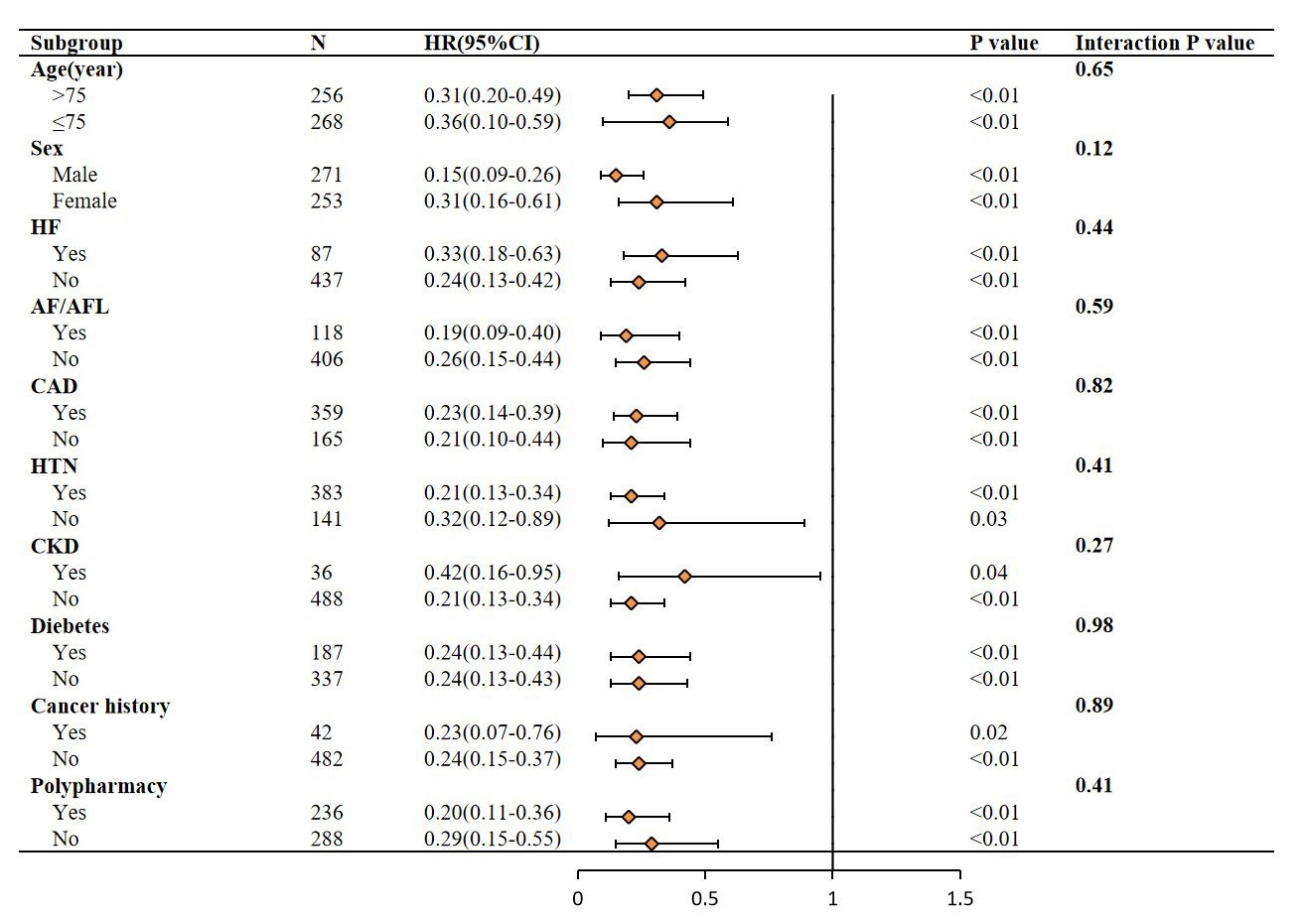
**Subgroup analysis forest plot for the association between IC score ≥7 and 5-year all-cause mortality**.

We employed Cox regression analysis to investigate the impact of IC score ≥7 on 5-year all-cause mortality across various subgroups. As outlined in the Methods section, the subgroups were classified based on age, sex, HF, AF/AFL, CAD, HTN, CKD, diabetes, cancer history and polypharmacy status. The results indicated that a higher IC score was associated with a reduced risk of 5-year all-cause mortality in all subgroups (all *p* < 0.05 for effects within each subgroup). No significant difference between groups was observed in subgroups (all *p* for interaction >0.05) (Fig. 5).

### 3.4 Comparison Between IC Score and the ACEF Score

To demonstrate the clinical value of IC score, we first compared the predictive performance of two Cox proportional hazards models: Model 1, which included the ACEF score, and Model 2, which included both the ACEF score and IC score. The C-index for Model 1 (the ACEF score) was 0.726, with a 95% CI ranging from 0.644 to 0.807. In contrast, Model 2 (ACEF + IC score) demonstrated an improved C-index of 0.789, with a 95% CI between 0.700 and 0.878. The difference in C-index between the two models was 0.063 (95% CI: 0.026–0.100), which was statistically significant (*p* < 0.01), indicating that the inclusion of the IC score enhanced the model’s discriminatory ability.

**Fig. 6.**
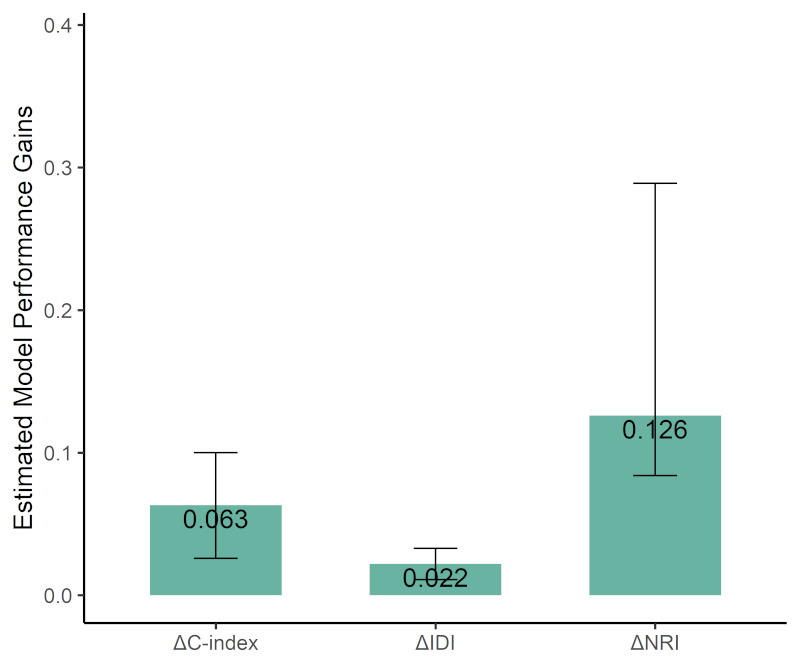
**Performance improvement of IC score compared to ACEF score across C-index, IDI, and NRI**. Abbreviations: IDI, integrated discrimination improvement; NRI, Net Reclassification Improvement.

We calculated the differences in IDI and NRI between the ACEF score and the IC score. The results from the IDI analysis indicated that compared to Model 1 (the ACEF score), Model 3 (IC score) provided improvement in model discrimination: 0.022 (95% CI: 0.011–0.033, *p* < 0.01). The results from the IDI analysis also showed that compared to Model 1 (the ACEF score), Model 3 (IC score) provided improvement in model discrimination: 0.126 (95% CI: 0.084–0.289, *p* < 0.01) (Fig. 6). Compared to the ACEF score, the IC score demonstrated improved discriminatory performance, as evidenced by higher IDI and NRI values statistically significant (both *p* < 0.01).

## 4. Discussion

In this prospective cohort analysis in elderly patients with cardiovascular disease, we observed that worse IC was strongly associated with increased risk of all-cause mortality. After adjustment for age, sex, comorbidity and lab tests, the observed associations remained robust, indicating a 1-point lower IC value was associated with a 26.6% increase in 5-year all-cause mortality.

Previous studies have demonstrated a strong association between declines in IC and adverse outcomes among older adults; however, these studies have primarily focused on the general population of community-dwelling older adults [5, 6, 18, 19]. In recent years, researchers have increasingly focused on assessing IC in specific patient populations, such as older adults with respiratory diseases. Evidence suggests that reduced IC is associated with a higher risk of 6-month rehospitalization in older patients with lower respiratory tract infections [20], as well as an increased 10-year all-cause mortality among those with respiratory diseases [21]. This raises the hypothesis that the assessment of IC may likewise be applicable to older adults with cardiovascular diseases, potentially serving as a tool for risk prediction and individualized management. To date, there is limited research on the association between IC assessment and prognosis in patients with cardiovascular disease. A study analyzed data from 443,130 participants in the UK Biobank and found that a decline in IC was associated with an increased risk of cardiovascular disease and cardiovascular mortality [22]. The findings of this study suggest that a decline in IC is associated with an increased risk of 5-year all-cause mortality in older adults with cardiovascular diseases. Furthermore, subgroup analyses and comparisons with the ACEF score demonstrated that IC score may serve as a useful tool for predicting clinical outcomes in older patients with cardiovascular diseases.

Lu WH *et al*. [23] found that elevated levels of inflammation-related biomarkers in plasma, such as growth differentiation factor 15 (GDF-15), are associated with lower levels of IC and rapid declines in IC among the elderly. Age-related chronic inflammation increases the risk of cardiovascular diseases [24], with elevated GDF-15 levels indicating poor cardiovascular outcomes [25]. This suggests that IC impairment may share underlying pathophysiological mechanisms with cardiovascular diseases. Thus, we believe that the assessment and intervention of IC hold substantial potential for application in the field of cardiovascular diseases, although current research in this area is limited.

The lack of standardized criteria for assessing IC is a major challenge hindering its wide use in clinical setting. Assessment methodologies for the five IC dimensions vary markedly across studies, and a universally accepted strategy for calculating an integrated global score remains lacking [8]. The general principle of IC assessment is to comprehensively evaluate the patient’s performance across five domains—locomotion, vitality, sensory, cognition, and psychological well-being. This approach is currently widely accepted by the majority of experts [8]. Our study is one of the studies that evaluating IC using the scoring system proposed by López-Ortiz S *et al*. [9]. At the same time, our study made minor modifications to the original scoring criteria; for instance, we used the GDS-5 and HADS-A scales for assessment in the psychological dimension and the MNA-SF in the vitality dimension. Although modifications were made, prior evidence supports the use of the assessment tools employed in this study as components of IC assessment or as instruments for prognostic assessment in patients with cardiovascular diseases. GDS-5 [26, 27], HADS-A [28], and MNA-SF [29] have all been utilized in previous studies to assess the prognosis of patients with cardiovascular diseases. Additionally, some studies have developed the IC score based on GDS-5 [30] and MNA-SF [31]. This indicates that our minor revisions to the IC scoring criteria are both scientifically sound and practically feasible. We believe that this IC score criteria is concise and clear, making it more suitable for clinical settings. Our study also validated the value of IC score in predicting clinical outcomes and calculated the thresholds for predicting all-cause mortality risk, providing new insights for the early identification of high-risk cardiovascular patients.

IC not only supports the prediction of disease prognosis but can also be utilized to develop personalized rehabilitation and intervention strategies. According to the “Integrated Care for Older People” (ICOPE) concept, multi-domain interventions for the elderly, including physical exercise, cognitive training, psychological support, nutritional guidance, and lifestyle improvements, can lead to significant improvements in cognitive dimension, psychological dimension, and locomotion dimension [32]. Exercise training for cardiovascular disease patients should avoid exercise-induced potential cardiac events. Therefore, comprehensive care for elderly cardiovascular patients requires collaboration between cardiologists and geriatric care rehabilitation teams [33].

Our study is among the first prospective cohort studies to demonstrate that IC score can independently predict 5-year all-cause mortality in elderly patients with cardiovascular disease. Furthermore, we developed a clinically practical IC scoring system tailored for this population, based on validated tools. Finally, our findings suggest that IC assessment can support not only prognostic prediction but also individualized intervention strategies, aligning with the ICOPE.

Our study has some limitations. First, as a single-center study, this research has a relatively small sample size and is limited to patients from the cardiology departments of tertiary hospitals, which may affect the generalizability of the results. Second, this cohort was not specifically established to study the association between IC and mortality. Although a multivariate Cox regression model was employed to control for potential confounders, the possibility of residual or unmeasured confounding cannot be entirely excluded. Third, this study assessed patients’ IC only during hospitalization and did not re-evaluate their IC during follow-up. Previous research has shown that changes in IC trajectories among older adults are important predictors of clinical outcomes [6, 34], highlighting a crucial direction for future research. Future research should explore how to integrate IC assessment with big data and artificial intelligence technologies to develop intelligent health management platforms, further advancing comprehensive elderly care based on monitoring and maintaining IC.

## 5. Conclusions

This study demonstrates that IC, as a comprehensive measure of an individual’s physical and mental reserves, is independently associated with 5-year all-cause mortality among elderly patients with cardiovascular diseases. A lower IC score was significantly linked to increased mortality risk, highlighting its prognostic value beyond traditional risk factors. The IC score, therefore, holds promise as a practical and integrative tool for early identification of high-risk individuals, enabling timely interventions. Given its multidimensional nature, IC score may facilitate more personalized and holistic clinical decision-making, especially when incorporated into routine care for elderly cardiovascular diseases patients. Future studies with larger, multi-center cohorts and standardized assessment protocols are warranted to validate these findings and further explore the utility of IC in guiding long-term management and rehabilitation strategies in geriatric cardiology.

## Availability of Data and Materials

Data are available upon reasonable request with the corresponding author.
